# Network analysis of an *in vitro* model of androgen-resistance in prostate cancer

**DOI:** 10.1186/s12885-015-1884-7

**Published:** 2015-11-10

**Authors:** Sujitra Detchokul, Aparna Elangovan, Edmund J. Crampin, Melissa J. Davis, Albert G. Frauman

**Affiliations:** 1 Clinical Pharmacology and Therapeutics, Department of Medicine, The University of Melbourne, Austin Health, Heidelberg, VIC Australia; 2 Systems Biology Laboratory, Melbourne School of Engineering, The University of Melbourne, Parkville, VIC Australia; 3 School of Mathematics & Statistics, The University of Melbourne, Parkville, VIC Australia; 4 School of Medicine, University of Melbourne, Parkville, VIC Australia; 5 ARC Centre of Excellence in Convergent Bio-Nano Science and Technology, The University of Melbourne, Parkville, VIC Australia

**Keywords:** Castrate resistant prostate cancer, Prostate cancer, Network based analysis, Protein-protein interaction, Hormone refractory, Steroid hormone receptors, Progesterone receptor

## Abstract

**Background:**

The development of androgen resistance is a major limitation to androgen deprivation treatment in prostate cancer. We have developed an *in vitro* model of androgen-resistance to characterise molecular changes occurring as androgen resistance evolves over time. Our aim is to understand biological network profiles of transcriptomic changes occurring during the transition to androgen-resistance and to validate these changes between our *in vitro* model and clinical datasets (paired samples before and after androgen-deprivation therapy of patients with advanced prostate cancer).

**Methods:**

We established an androgen-independent subline from LNCaP cells by prolonged exposure to androgen-deprivation. We examined phenotypic profiles and performed RNA-sequencing. The reads generated were compared to human clinical samples and were analysed using differential expression, pathway analysis and protein-protein interaction networks.

**Results:**

After 24 weeks of androgen-deprivation, LNCaP cells had increased proliferative and invasive behaviour compared to parental LNCaP, and its growth was no longer responsive to androgen. We identified key genes and pathways that overlap between our cell line and clinical RNA sequencing datasets and analysed the overlapping protein-protein interaction network that shared the same pattern of behaviour in both datasets. Mechanisms bypassing androgen receptor signalling pathways are significantly enriched. Several steroid hormone receptors are differentially expressed in both datasets. In particular, the progesterone receptor is significantly differentially expressed and is part of the interaction network disrupted in both datasets. Other signalling pathways commonly altered in prostate cancer, MAPK and PI3K-Akt pathways, are significantly enriched in both datasets.

**Conclusions:**

The overlap between the human and cell-line differential expression profiles and protein networks was statistically significant showing that the cell-line model reproduces molecular patterns observed in clinical castrate resistant prostate cancer samples, making this cell line a useful tool in understanding castrate resistant prostate cancer. Pathway analysis revealed similar patterns of enriched pathways from differentially expressed genes of both human clinical and cell line datasets. Our analysis revealed several potential mechanisms and network interactions, including cooperative behaviours of other nuclear receptors, in particular the subfamily of steroid hormone receptors such as PGR and alteration to gene expression in both the MAPK and PI3K-Akt signalling pathways.

**Electronic supplementary material:**

The online version of this article (doi:10.1186/s12885-015-1884-7) contains supplementary material, which is available to authorized users.

## Background

Prostate cancer (PCa) is among the most commonly diagnosed diseases in males, and remains a leading cause of death in developed countries [1]. In Australia, PCa is the most commonly diagnosed cancer and accounted for approximately 13 % of all cancer-related deaths in males in 2010 [2, 3]. PCa tumour growth is initially dependent on androgens as documented by Huggins as early as 1941 [4], making androgen deprivation therapy (ADT) the first line treatment. However patients often ultimately develop an androgen independent state of PCa, often referred to as Castrate Resistant Prostate Cancer (CRPC) and there are no effective treatments for this state of PCa.

Androgens act through the androgen receptor (AR) signalling pathway. A review by Feldman [5] details five broad mechanisms through which PCa cells can survive despite low levels of serum testosterone in CRPC. Three out of the five mechanisms involve AR signalling, where in the absence of serum testosterone, AR continues to play an active role in CRPC through adrenal testosterone, increased AR expression level (AR amplification), AR mutation where other steroid hormones (such as progesterone or oestrogen) or mutated co-regulators activate AR and “outlaw” AR where AR becomes ligand independent, for example through alternative splicing. The other two mechanisms bypass AR altogether, and CRPC cells survive through alternative pathways, such as through up-regulation of oncogenes that block signals for cell apoptosis and cause cell proliferation.

The establishment of an *in vitro* model of CRPC is crucial for the study of the progression into advanced stage PCa. Previous studies have used the androgen-sensitive cell line LNCaP in long-term culture in androgen deprived conditions. These long-term cultures were carried out ranging from 2 months up to 24 months during which time androgen resistance develops in these cells [6–9].

The question remains whether these *in vitro* studies reflect biological features in human tumours, a question addressed in this current work. In our experiments, we study the various CRPC mechanisms using existing human tumour datasets [10] and *in vitro* LNCaP cell line model through computational methods of RNA sequencing expression, differential expression and network analysis. Data from transcriptomic profiling of patients [10] receiving ADT (LHRH agonists with anti-androgen flare protection [11]) for approximately 22 weeks were compared with our cell line model to determine what molecular changes were common to the two datasets, and to establish the suitability of our model system for studying drivers of developing androgen insensitivity in vivo.

## Methods

### LNCaP cell line and reagents

The human PCa cell line LNCaP was obtained from American Type Culture Collection (ATCC) (Manassas, Virginia, USA). Cells were maintained and propagated as monolayer cultures in RPMI 1640 medium (Life Technologies Corporation) with 10 % foetal bovine serum (FBS) (Thermo Scientific), and 100 units/mL penicillin and 100 μg/mL streptomycin (Life Technologies Corporation).

### *In vitro* androgen independent model

**Fig. 1:**
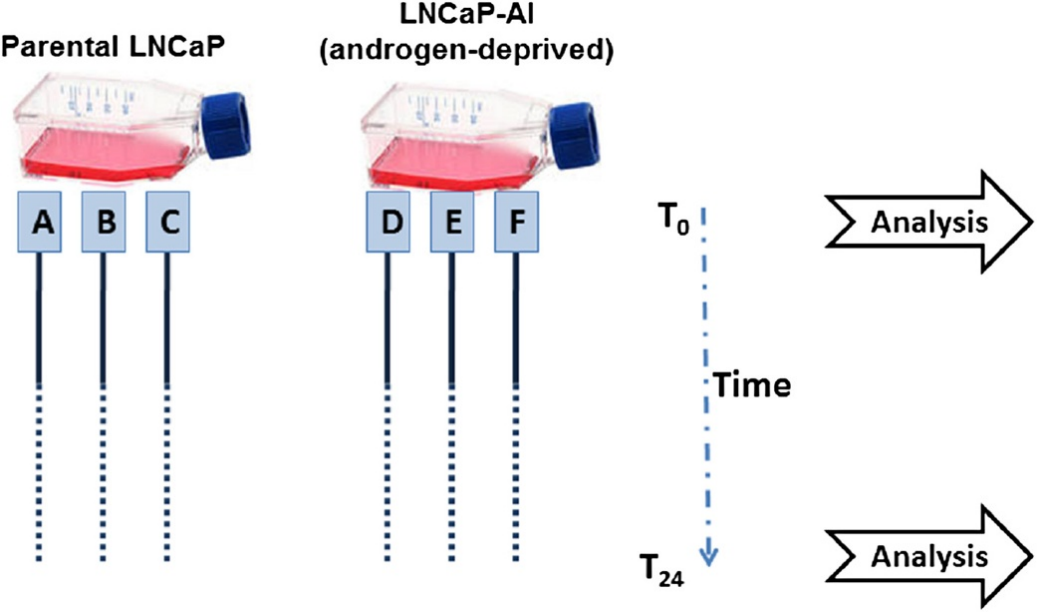
*In vitro* CRPC cells workflow. All conditions are replicated in triplicate so that each condition has three independent biological replicates

*In vitro* CRPC models were established by prolonged cultures of androgen-sensitive LNCaP cells (parental). We have generated cells grown under (i) a control condition for parental LNCaP, in FBS; (ii) media with charcoal-stripped FBS which removes low molecular weight hormones including steroid, thyroid and peptide hormones (CS-FBS,  androgen-deprived) (Fig. 1). Cells that were grown in androgen-deprived condition are referred to as LNCaP androgen independent (LNCaP AI) cells.

### Cell viability assay

Trypan blue dye exclusion was performed to examine cell viability of cell lines. Routine cell harvesting was performed and cell suspension was diluted (1:1) with 0.1 % (w/v) trypan blue dye (Sigma Aldrich) in dH2O and transferred (20 μL) to a haemocytometer for counting, using an inverted microscope (Model CK2, Olympus Optical Co. Ltd, Japan). Total of viable and non-viable cell numbers were counted by trypan blue dye exclusion.

### Cell proliferation assay

Relative cell numbers were measured by 3-(4,5-Dimethylthiazol-2-yl)-2,5-diphenyltetrazolium bromide or MTT colorimetric assays. Parental cells were grown in phenol-red free CS-FBS media 3 days prior to the start of the experiments. Cells were plated on 96-well plates in phenol-red free media + CS-FBS. After overnight attachment, cells were treated with 1–10 nM DHT or 1–10 μM bicalutamide for 6 days. Cell proliferation was examined by addition of MTT to the assay plate and the absorbance read at 590 nm, reference filter 620 nm.

### RNA isolation

RNA was isolated from cells in triplicate using the Rneasy Mini Kit (Qiagen Pty Ltd.) according to manufacturer’s instructions. RNA quantity was assessed using a NanoDrop 2000 UV–vis Spectrophotometer (Thermo Scientific) at A260nm and RNA integrity was determined using the A260nm/A280nm ratio. For RNA sequencing, RNA was checked for yield and quality using an Agilent 2100 Bioanalyzer (Agilent Technologies, Inc.).

### Reverse-transcript PCR and quantitative PCR (qPCR)

RNA was extracted from parental LNCaP and subline cells as described above. cDNA synthesis were performed using M-MuL-V kit (Life Technologies Corporation). cDNA samples were then analysed using AR (assay ID Hs00171172_m1) and 18S (assay ID Hs99999901_s1) (as control) Taqman gene expression assays (Applied Biosystems, Life Technologies Corporation). PCR amplification was performed in a 25 μL final volume (total 54 ng cDNA per reaction) using 7500 Real-time PCR System (Applied Biosystems, Life Technologies Corporation). mRNA expression of AR was normalized in relation to the control 18S expression. Data are expressed as fold difference to parental LNCaP cell line.

### Lysate extraction and western blotting

Modified radioimmunoprecipitation (RIPA) buffer was used to extract proteins from the cell. Medium was removed from cells and cells were washed twice with ice-cold PBS before addition of ice-cold RIPA buffer containing 1× Complete Mini EDTA-free protease inhibitor tablet (Roche Diagnostics). Protein concentration of the whole cell lysates was determined using the Bradford assay [12]. Proteins were separated by SDS-PAGE. Protein bands were then transferred to nitrocellulose paper and incubated with 1/200 diluted AR antibody (N-20) [Santa Cruz Biotechnology] and peroxidase conjugated antibody respectively. Peroxidase linked antibody was purchased from Amersham™ (GE Healthcare Biosciences). β-actin levels were used as a loading control. Protein bands were visualized after chemiluminescent reaction.

### Immunocytochemistry

#### Preparation of cover slips

Cover slips were positioned in a sterile beaker and were immersed in ice-cold 100 % (v/v) methanol under aseptic conditions. The beaker was placed in a container filled with ice and left in the fume hood under UV light for 2 h to sterilise. The cover slips were allowed to dry and were then placed into each well of the 6-well plate.

Cells were passaged by trypsinisation. Cell suspensions were added to prepared 6-well plate (with cover slip in each well) at a concentration of 1 × 10^5^ cells/well. Cells were allowed to grow at 37 °C/5 % CO_2_ in a humidified incubator to a confluence of 50–70 %, with addition of fresh media if needed.

#### Immunostaining

When cells had reached confluence, the old media were aspirated from each well. Coverslips were washed with PBS buffer for 5 min and then were fixed in ice-cold 100 % (v/v) methanol for 10 min at room temperature. Cells were permeabilised in PBS containing 0.1 % (v/v) Triton-X 100 for 5 min. The cover slips were washed twice with PBS for 5 min. Immunohistochemical analysis was performed using the labelled streptavidin/biotin-based LSAB + ™ (Labeled Streptavidin Biotin) system/HRP kit (DAKO Australia) according to the manufacturer’s protocol, with minor alterations. All incubations were carried out at room temperature. Primary antibody monoclonal mouse anti-human PGR antibody (clone A9621A, R&D systems) at working concentration of 1 ug/mL in an antibody diluent solution (DAKO Australia) was applied to each slide for 1 hour. This was followed by sequential incubation of fixed cells with anti-goat/rabbit/mouse biotinylated-link antibody and peroxidase-labelled streptavidin. PGR protein expression was visualised by incubation with 3,3’-diaminobenzidine chromogen solution (DAB+ substrate buffer), yielding a brown end-product. Fixed cells were counterstained with haematoxylin. As a negative control, the fixed cells were incubated with isotype antibody IgG_2a_.

### Cell migration and invasion assays

Migration assays were performed in BIOCOAT™ control microporous membrane filter inserts while invasion assays were performed on Matrigel matrix-treated polyethylene terephthalate (PET) membrane filter inserts in 24-well tissue culture plates (BD Biosciences, Australia), as described previously [13]. Briefly, the BIOCOAT™ inserts are 6.4 mm in diameter, and the pore size on the membrane is 8 μm. Cells were washed once with versene, detached at 80–90 % confluence with 0.05 % trypsin/EDTA, and resuspended in serum-free media. The inserts were incubated with serum-free media at 37 °C for 2 h to rehydrate. Media containing 10 % FCS (as a chemoattractant) was added to the lower wells and a 500 μL cell suspension was added to the inserts at a density of 5 × 10^4^ cells/insert. Migration across the membrane was allowed to proceed at 37 °C 5 % CO_2_ for 48 hours. Cells that did not migrate through the membrane were removed using cotton swabs, and cells that migrated through the membrane filters were fixed with 100 % v/v methanol and stained with 0.05 % v/v crystal violet dye (Sigma-Aldrich, Australia). The membranes were carefully removed from the insert using a scalpel blade and mounted onto glass slides. The number of migrated cells per insert (10 fields were chosen from each insert) was counted using the M2 program of the Micro-Computer Imaging Device-assisted image analysis program (MCID, Imaging Research, Inc., St. Catharine’s; Ontario, Canada) under light microscopy at magnification ×200. All experiments were repeated in triplicate on each of three separate occasions.

### LNCaP RNA sequencing

LNCaP and LNCaP-AI RNA samples were sequenced on an Illumina platform (Illumina HiSeq2000) by the Australian Genome Research Facility (AGRF), Melbourne, Australia. CASAVA1.8 pipeline was used to generate the sequence data. The sequence reads were processed through a quality control pipeline (FastQC, SolexaQA) (investigating quality matrices such as presence of ambiguous bases, adaptor contamination, PCR duplicates, GC content and sequence complexity) with required quality score > Q30 for all reads.

### Human clinical data

The RNA reads from 7 patients taken before (Hormone naïve) and after (Hormone resistant, defined as two consecutive rise of PSA more than 10 %) androgen-deprivation treatment (ADT) (approximately 22 weeks) were obtained from a study conducted by Rajan et al. [10] on the effects of ADT on advanced PCa. mRNA reads, obtained by next gen sequencing techniques were reanalysed using the protocol described below.

### Computational methods

We have used standard methods for RNA sequencing differential expression analysis. Tophat v2.0.9 [14] was used to align the RNA sequencing reads using hg19 as the reference genome and EdgeR v3.4.2 [15] was used for differential expression analysis.

For our protein to protein network analysis, we obtained the network for homo sapiens from PINA2 interaction resource [16]. The gene, UBC, which has >5000 interactions in this dataset was removed from the analysis. We constructed a protein interaction network by taking the set of genes differentially expressed in both datasets, and collecting all protein interactions involving the products of these genes by querying the human interactome with Uniprot Accession numbers obtained from Biomart.org. The resulting network was pruned to remove proteins with a degree of 1, such that a protein not in our original list of commonly differentially expressed genes was only retained in the network if it interacted with at least two proteins encoded by the query set.

We also used machine learning to predict gene regulatory network using GENIE3 [17]. GENIE3 uses random forests for regression trees to compute an importance measure of the relationship between the predictor variables and the output variable. Modelling gene regulatory network inference as a machine learning problem, the expression level of each gene, computed using cuffnorm [14], is the output variable and the expression levels of transcription factors are the predictor variables.

All statistical tests were carried out in R using the hypergeometric distribution test function.

## Results

### Characterisation of androgen-deprived LNCaP subline cells (LNCaP-AI)

**Fig. 2:**
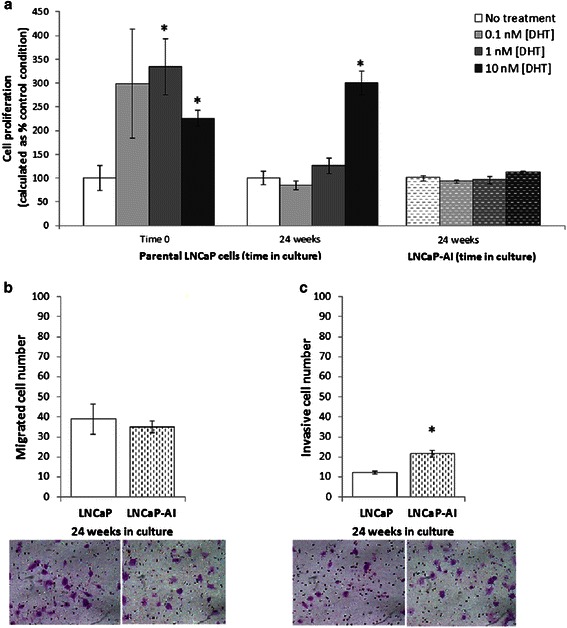
Androgen responsiveness of LNCaP and LNCaP-AI cells. **a** Androgen responsiveness of LNCaP-AI cells was lost by 24 weeks of prolonged culture compared to parental LNCaP cells. Data are shown as % cell proliferation of DHT treated vs. no DHT treatment condition. For this proliferation assay, cells were treated with different concentrations of DHT for a period of 6 days. **b** No difference in cell migration was seen between parental and subline cells. However, LNCaP-AI cells were more invasive (**c**) compared to parental LNCaP cells. (*N* = 3, error bars = SEM, * indicates *P* < 0.05). Picture inset underneath the graphs are representative images of cell migration and invasion (cells are stained purple)

LNCaP cells were grown in culture under androgen-deprived conditions for 24 weeks (LNCaP-AI). The viability of cells in culture was examined regularly and LNCaP-AI cells initially showed poor growth and proliferation after growth in androgen-deprived culture. Over a period of time, however, cells adapted and started to grow vigorously (data not shown). Dihydrotestosterone (DHT) dose–response stimulation of cell proliferation was performed for parental LNCaP and LNCaP-AI after prolonged culture of 24 weeks to determine androgen responsiveness. These assays established that LNCaP-AI cells lost androgen-responsiveness after 24 weeks of culture when compared to parental LNCaP cells that were grown in parallel, DHT (concentration from 0.1 to 10 nM) not being able to stimulate increased proliferation in LNCaP-AI (Fig. 2a).

### AR expression and activation

**Fig. 3:**
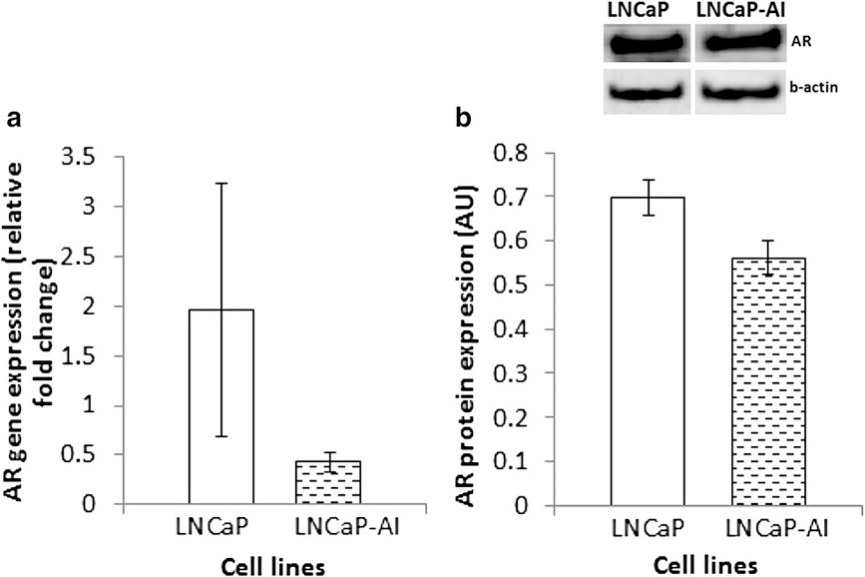
AR expression in LNCaP and LNCaP-AI. **a** AR gene and **b** Protein expression (densitometric analysis of AR corrected for β-actin expressed in AU [arbitrary units]) of LNCaP and LNCaP-AI cells at 24 weeks of culture. (*N* = 3, error bars = SEM)

We examined other phenotypic characteristics and observed that LNCaP-AI cells also displayed increased cell invasion after 24 weeks of prolonged culture compared to parental control cells, using Boyden chamber cell invasion assays (Fig. 2b and c), consistent with a more aggressive phenotype. AR gene (qPCR) and protein (Western Blot) (Fig. 3a and b, respectively) expression in LNCaP-AI cells was not statistically significantly (*P* > 0.05) different to parental LNCaP cells. This is consistent with the fact that increased AR expression is not the sole determinant of initiation of PCa or development of hormone refractory PCa [18].

Given that our cell line model demonstrates a phenotype consistent with androgen insensitivity after 24 weeks of growth in androgen deprived culture conditions, we wished to establish how well our model reproduces the molecular phenotype seen in androgen resistant human tumours. To do this, we compared the results of our RNA sequencing of sensitive and insensitive cells, with a previously published study of gene expression in human tumours before and after treatment with androgen deprivation therapy. To ensure the results were comparable, we re-analysed the human data using the same analysis protocol we used for our own RNA sequencing data (see Methods section).

**Fig. 4:**
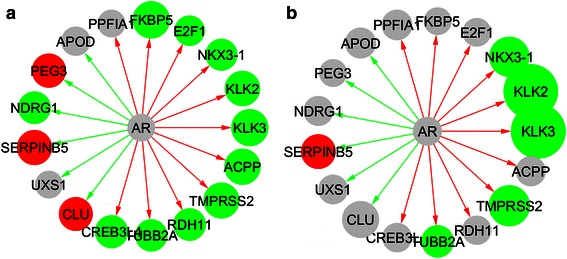
Expression levels of AR regulated target genes. Green nodes indicate under expressed genes, whereas red nodes represent over expressed genes; edges are coloured to indicate the expected direction of regulation (red edges indicate positive regulation, whereas green edges indicate inhibition). Genes shown in grey are not significantly differentially expressed. **a** AR regulated genes in human tumour samples; **b** AR regulated genes in LNCaP-AI cells

The AR was not differentially expressed in either the human tumour samples or the cell line model (Fig. 4). However genes, such as *KLK3* and *TMPRSS2* that are normally uniquely up regulated by AR are down regulated as shown in Fig. 4 suggesting that AR is not actively regulating these targets.

We also examined expressed AR Isoforms AR-001, AR-002, AR-003, AR-004 (AR-V7) [19], AR-005 and AR-201 to determine if differential expression of any of these isoforms might underpin the androgen insensitive phenotype in either our cell lines or the human tumours (Table 1). We found that none of the isoforms were significantly differentially expressed either after treatment in patient samples, or in our cell line model. Interestingly, several isoforms were detectable in human tumour data both before and after treatment, and in both time points of our experimental system, and transcripts show great variation in composition of functional domains (Table 1).

**Table 1:** Changes in the expression of individual isoforms of the Androgen Receptor in our cell line model and in the human tumour data. No significant differential expression is seen in either dataset. Transcripts are taken from the Ensembl AR Gene Transcript Table (ENSG00000169083)

Transcript Identifier	HGNC transcript name	Protein length	Domains	Cell Line Log Fold-Change	Cell Line *P*-Value	Tumour Log Fold-Change	Tumour *P*-Value
ENST00000374690	AR-001	920aa	NTD, ZF, LBD	0.03	0.95	0	1
ENST00000396043	AR-002	388aa	ZF, LBD	0	1	−0.11	0.92
ENST00000513847	AR-003^	-	-	0.72	1	0	1
ENST00000504326	AR-004 (AR-V7)	644aa	NTD, ZF	−1.38	0.57	1.82	0.69
ENST00000514029	AR-005*	600aa	NTD, ZF(partial)	−0.19	1	0	1
ENST00000396044	AR-201	734aa	ZF	0	1	−0.08	0.96
ENST00000612010	AR-202	642aa	NTD, ZF	ND	ND	ND	ND
ENST00000612452	AR-203	737aa	NTD (partial), ZF, LBD	ND	ND	ND	ND
ENST00000613054	AR-204	572aa	NTD	ND	ND	ND	ND

*Nonsense mediated decay; ^Processed transcript; *ND* not detected, *NTD* N-terminal domain, *ZF* zinc finger, *LBD* ligand binding domain. AR-004 is identified as the AR-V7 transcript based on the description of the isoform provided by Krause and colleagues (2014), as the protein encoded by this transcript aligns to the first 627aa of the full length AR, and contains 15 unique amino acids, with one overlapping a splice site [19]

### Common differentially expressed genes are found in human PCa and LNCaP models

We used a published clinical dataset [10] to determine whether our cell line model of androgen insensitivity displays molecular features in common with human disease. We found 213 genes were differentially expressed in both experiments. This highlights that while there are definite differences in the molecular phenotypes of our model and the human tumour data, our model none-the-less recapitulates molecular features found in advanced human disease. We also examined the pathways that are enriched for differentially expressed genes in each experiment, and found that differential expression in both datasets converged at the pathway level (full results Additional file 1: Table S1 and S2); in particular, two related pathways, MAPK and PI3K signalling are both strongly implicated by the differentially expressed genes of both datasets. Previous reports adopting disease-associated gene network and pathway analyses in PCa have revealed novel regulatory mechanisms and were more powerful than the analysis of gene expression level alone [20–24].

### Analysis of protein network overlap between in the human PCa and LNCaP models

**Fig. 5:**
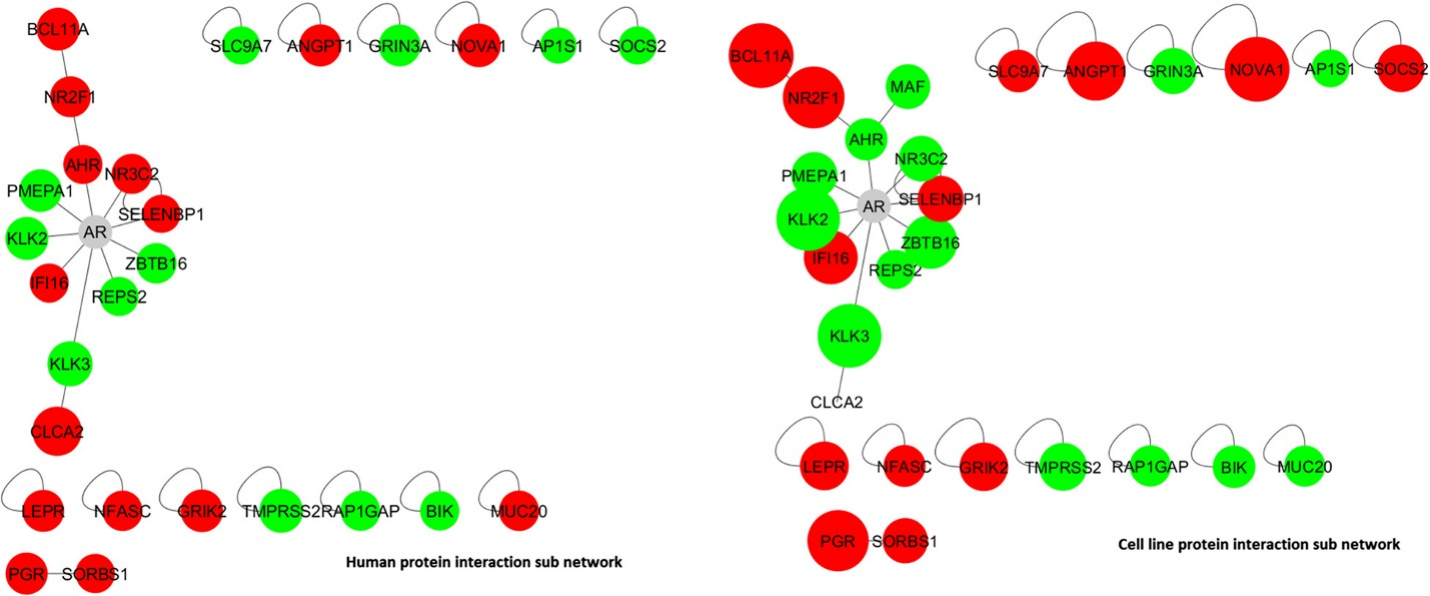
Protein interactions within the set of 213 genes differentially expressed in both experimental datasets. AR has been included in this network for reference. Red nodes represent genes with increased expression in the resistant state, whereas green genes have lower expression. AR itself is not differentially expressed

In order to explore the mechanisms captured in our cell line model and in common with those in the human tumour data, we focused on the set of 213 genes that were differentially expressed in both datasets and first examined protein interactions among the proteins encoded by these genes (Fig. 5). In particular, the presence of two up-regulated nuclear receptor transcription factors in this network (*PGR* and *NR2F1*) suggests that these transcription factors may play a role in the androgen insensitive state. As the functional effects of differential expression propagate through the interaction partners of proteins, we expanded our protein interaction network to include interaction partners of the 213 commonly differentially expressed genes in order to capture a broader set of proteins whose functions are likely to be affected by these changes. The initial network expansion added 1700 new interacting proteins, 80 % of which interacted with only one protein from our query set. We subsequently pruned this network in order to increase the likelihood of identifying proteins through which the functional effects of altered gene expression are likely to propagate: we removed proteins with a degree of 1 in this network, and insisted that all proteins interacted with at least two from our query set of 213. This resulted in a network of 492 proteins with 1062 interactions, which we refer to as our disrupted network. While genes differentially expressed in both experiments encode only 170 proteins in this network (as some of the 213 lack known interactions), a further 85 correspond to genes disrupted in either one or the other experiment, such that 45 % of all proteins captured in this network have evidence for significant differential expression in PCa either in human tumours or in our cell line model.

In order to determine the likely effects of this disrupted network on the development of androgen resistance in our model, we performed a functional analysis looking at enriched Gene Ontology terms and signalling pathways. Analysis of the molecular functions enriched in this network revealed two very strong functional signatures, related to transcription factor binding (58 genes, corrected P value 1.9 × 10^−19^, including steroid hormone receptor binding with a corrected p value of 4.2 × 10^−8^), and protein kinase activity (55 genes, corrected p value 2.7 × 10^−14^). This suggested that our disrupted network represents two broad adaptive mechanisms that may be at play in both our model and the human tumour data: alteration of transcriptional regulation in response to loss of AR regulation (seen in Fig. 4); and altered signalling driving proliferation and inhibiting cell death. To explore both these mechanisms, we (i) performed analysis of the regulatory impact of transcription factors, and (ii) performed pathway analysis of the disrupted network .

(i) *Regulatory impact of nuclear receptors in androgen insensitive tumours and cells*

In the human data, 15 of 48 known nuclear receptors are differentially expressed and of the 8 steroid hormone receptors, four are differentially expressed (Table 2). In our cell line data set, 11 nuclear receptors are differentially expressed (Table 2). It has previously been reported that nuclear receptors (NRs), particularly the sub family of steroid hormone receptors, with a similar structure and binding motif, may provide some functional redundancy. Thus, we hypothesize that other nuclear receptors may be up regulated in response to loss of AR signalling, and compensate for the loss of gene regulation by AR in androgen insensitive PCa.

**Table 2:**
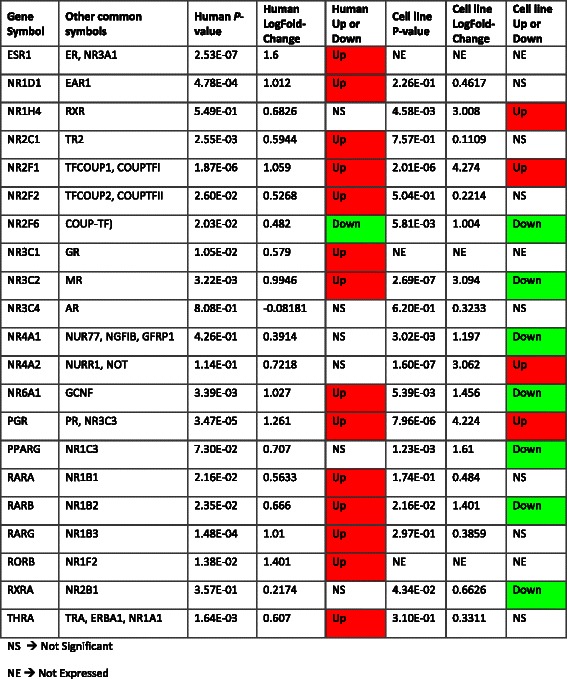
Differential expression of nuclear receptor transcription factors in human tumour and cell line model data

We performed a computational analysis to predict regulatory relationships between NRs and potential target genes. To do this, we use the method Genie3 [17], which uses random forests for regression trees to compute an importance measure for the relationship between the predictor variables (here, the expression level of NR transcription factors) and the output variables (expression levels of all other genes). Because individual predictions of association between transcription factors and targets based on expression data are likely to be noisy and error prone [25], we do not attempt to use these networks to infer mechanism; rather we look for strong patterns of shifting influence as captured by the broad-scale loss or gain of targets with high importance measures (the top ten transcription factors ranked by increasing size of inferred regulatory networks are shown for human tumour data in Table 3, and cell line data in Table 4).

**Table 3:** Regulatory importance of transcription factors in human tumour data

Nuclear Receptor	Network size before treatment	Network size after treatment	Increased influence post-treatment
PGR	28	114	86
ESRRA	2	77	75
PPARD	2	65	63
THRB	32	95	63
ESR2	29	67	38
NR2F6	4	37	33
NR4A2	5	35	30
ESRRG	21	41	20
RXRG	7	24	17
NR1I3	26	39	13

**Table 4:** Regulatory importance of transcription factors in cell line model of androgen insensitivity

Nuclear Receptor	Network size LNCaP cells	Network size LNCaP-AI cells	Increased Influence in LNCaP-AI
PGR	1	53	52
RXRA	3	52	49
RARG	2	44	42
RORA	0	30	30
NR4A3	1	29	28
NR5A2	0	27	27
ESR2	0	23	23
VDR	18	41	23
NR2C1	0	22	22
ESRRG	22	42	20

**Fig. 6:**
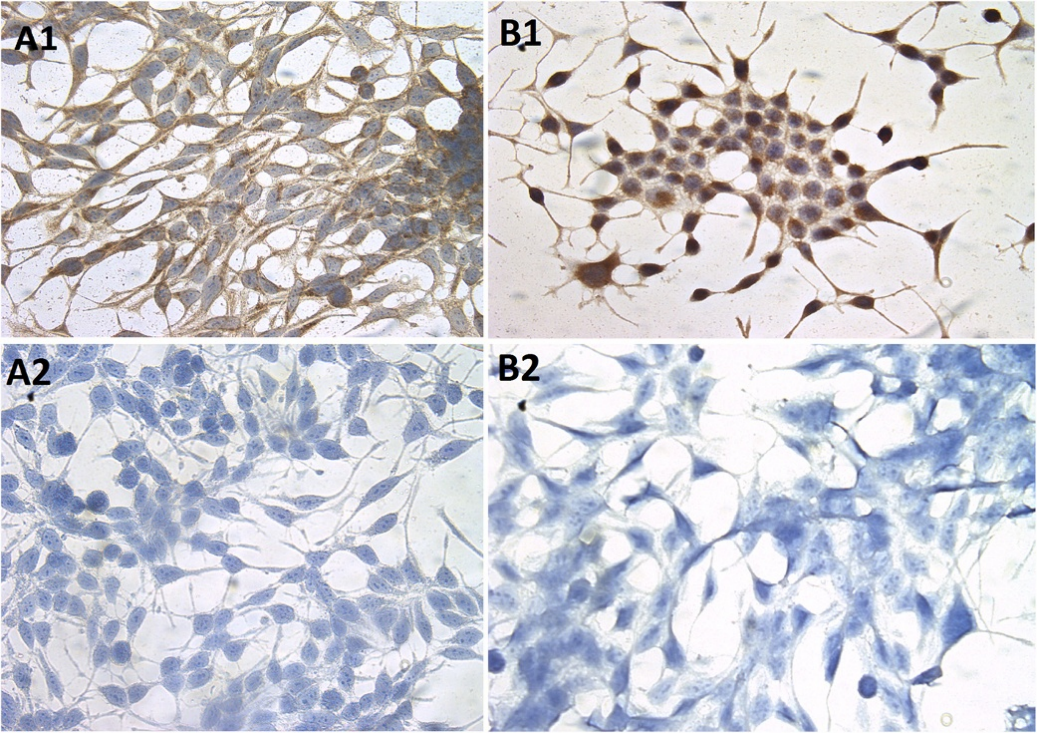
PGR protein expression in prostate cancer cell line, LNCaP (Panel **a**) and LNCaP-AI (Panel **b**). Cells were grown, fixed and then stained for PGR (*A1, B1*) and control antibody (*A2, B2*). The localisation of PGR was more predominant in LNCaP-AI than LNCaP in the cytoplasm. PGR expression in the Nucleus is also more prominent in LNCaP-AI cells. Original magnification ×400

Most notably, the progesterone receptor (PGR), showed the largest increase in inferred network size of all nuclear receptors in both the human and cell line data (we confirmed PGR protein expression via immunocytochemistry (Fig. 6)), suggesting that this transcription factor may be assuming a regulatory role that in part compensates for the loss of AR regulation. It should be noted that these networks are derived from relationships in the expression data, and are not influenced by the size of the interaction networks available for these transcription factors.

(ii) *Pathway analysis of the disrupted network*

We observed a strong enrichment for protein kinase function in our disrupted network, hinting at adaptive mechanisms operating through signalling pathways to promote proliferation in our androgen insensitive cells. We mapped differentially expressed genes from our disrupted network to KEGG signalling pathways in order to identify mechanisms through which these gene expression changes may affect cellular phenotype. Notably, MAPK and PI3K-Akt signalling both showed a large number of differentially expressed genes (MAPK - cell line 42, human 58; and PI3K-Akt - cell line 65, human 92). Within these pathways, we see some striking patterns in shared disruption. In PI3K-Akt signalling, for example, reactions leading to cell survival, growth and proliferation outcomes are up-regulated in both human and cell line data (see Additional file 1: Figure S2 and S4 where these data are mapped to KEGG pathways). Similarly in MAPK signalling, there is a suggestion of enhanced signalling through to NF-ƙB and c-JUN, which could have an impact on proliferation and anti-apoptotic regulation [26] (see Additional file 1: Figure S1 and S3). Interestingly, both human and cell data indicate that signalling through the MEK-ERK module itself may actually be reduced through a combination of down regulation of MEK and up-regulation of inhibitors of ERK. While this may seem counter intuitive, a recent paper characterising the phosphoproteomic changes in PCa found that signalling through ERK was in fact reduced in androgen-independent PCa [27], consistent with our findings here.

## Discussion

Here, we have described an *in vitro* model of the development of androgen insensitivity in PCa, and compared the characteristics of our cell line model to that of human disease. This cell line model is described here.

### Role of AR signalling pathway

In our experiments, we found that AR is not differentially expressed and genes (such as *KLK3* and *TMPRSS2*) that are uniquely up regulated by AR are all down regulated in both the human PCa tumour cells and the LNCaP model as shown Fig. 4. Genes such as *CLU*, *PEG3* etc. that are suppressed by AR are up regulated (except for *NDRG1*) in human PCa cells, and in the LNCaP model. The difference in AR gene and protein (Fig. 3) expression in LNCaP-AI cells was also not in itself statistically significant, and these results are consistent with loss of AR signalling in both systems.

Clinically, rising levels of prostate specific antigen (PSA) show dependence on AR in CRPC and treatments include directly targeting AR, using AR antagonists [28, 29]. However not all patients respond to AR antagonists, and those who do tend to relapse [28]. This shows that tumour cells in CRPC can continue to survive, bypassing the AR signalling pathway. In our experiment, AR is not active in the LNCaP model and the transcription abundance changes in both tumour and cell line data (Fig. 4) suggest that in at least the human data we compare with here, AR is not actively regulating its normal targets. Alternative pathways bypassing AR signalling are therefore likely to be responsible for the development of androgen insensitivity in our cell line model. This emphasises the clinical relevance of the model we have developed for understanding CRPC that is driven by these kinds of resistance mechanisms. Other genes that have been shown to negatively regulate AR and AR signalling are overexpressed in our experimental model, including Id1 [30] and IFI6 [31]. While these are not likely to be the only mechanisms driving clinical CRPC, a model such as ours that allows for the longitudinal study of these processes will provide valuable insight into CRPC driven by such bypass mechanisms [32].

### Role of nuclear receptor super family and the steroid hormone receptor (SHR) subfamily

AR belongs to the steroid receptor family within the nuclear receptor superfamily. Of the 48 nuclear receptors [33] known in the human genome, the sub family of steroid hormone receptors contains *ER*s (*Nr3A1/ESR1*, *NR3A2/ESR2*), *ERR*s (*ESRRA/NR3b1*, *NR3b2*), *GR* (*Nr2c1*), *MR* (*Nr3c2*), *PR*s (*NR3C3*), and *AR* (*NR3C4*) [34]. DNA binding domain sequence for AR is very similar to PGR and glucocorticoid receptor (GR) [35], so these genes, that are structurally similar to AR, may be replacing AR regulation of some genes. Given almost all classes of SHR are differentially expressed in our samples (Table 2), a possible explanation is that in the absence of AR activation the other SHRs compensate and become active and are involved in CRPC cell multiplication. GR has already been implicated in CRPC bypassing AR blockade [36] and GR is up regulated in the human data set (Table 2).

In our experiments *PGR* is differentially expressed and up-regulated (see Table 2) and is also part of the top-scoring protein network (see Fig. 5). Recently, the analysis of samples from over 500 PCa patients who have never been treated with hormone therapy revealed that PGR expression in both areas of prostate tumour epithelium and tumour stroma is an independent prognostic factor of clinical failure [37]. Previously, it has been found that PGR has a negative correlation with AR in hormone refractory PCa [38]. PGR was also found to be increased in CRPC but decreased in localised PCa, although the findings did not reach statistical significance [38]. Our finding reflects this clinical finding that PGR is upregulated in androgen-deprived cells and was expressed negatively to AR. As an oestrogen-regulated gene [39], PGR expression could also be interrelated to the importance of oestrogen receptors (ER) in PCa. The two major subtypes of ER, ERα and ERβ have been previously investigated in PCa [40–42]. The interchanging role between ERβ down-regulation in hormone naïve PCa and ERβ up-regulation in CRPC in matched samples has been documented [43]. Other researchers also speculated that ERβ expression might be mediated by phosphorylated AR at serine 210 (_p_AR(210)) [41, 43]. Although the role of circulating oestrogen in PCa carcinogenesis is still controversial [44], it may be worth investigating the role of ER and PGR in CRPC. Research into the role of PGR and PCa is still in its early stage and also, the lack of available antibody for all isoforms of PGR hinders advancement in the field. While ER expression is not enriched in our network analysis, their associated relationship with PGR compels the need for further investigation of both nuclear receptors in future study.

NR2F1 and NR2F2, also known as COUP-TFs, are orphan nuclear receptors and they occupy consensus DNA binding sites shared by other nuclear receptors including thyroid hormone receptor, retinoic acid receptor, oestrogen receptor and AR [45–47]. This allows COUP-TFs to modulate transcriptional activities of these nuclear receptors. As well, COUP-TFs are able to exert regulatory control via direct competitive binding to coactivators and corepressors of these nuclear receptors [48]. Although more is known about the role of NR2F2 in PCa [45, 49], both *COUP-TF* genes, *NR2F1* and *NR2F2* display nearly 100 % homology in their DNA-binding domain (DBD) and ligand- binding domain (LBD), though no natural ligand has been identified for these COUP-TFs [50]. NR2F2 was found to promote prostate tumourigenesis via inactivation of TGF-β signalling in PTEN-null mice [49]. In contrast, another study found NR2F2 to inhibit PCa cell growth via direct binding to AR preventing androgen-dependent signalling [45]. A previous study has shown that NR2F1 expression was negatively regulated by AR but in the presence of AR antagonist, this gene was positively regulated [51]. It can be speculated that in our model long term growth in androgen-deprived conditions decreases androgen-dependent AR-signalling, and could cause up-regulation of the *NR2F1* gene. This may suggest that functions of these COUP-TFs depend on the tumour microenvironment and expression profiles of other nuclear receptors and transcription factors [52]. A balance between the two COUP-TFs may also be important in the control of CRPC progression and further longitudinal studies will be important for determining the shift between these two receptors in CRPC.

### AR signalling bypass through MAPK and PI3K-AKT signalling pathways

An effective AR signalling bypass would be through genes that support proliferation and inhibit apoptosis, even in the absence of androgens and AR [5]. In our experiment, there are a number of genes that are differentially expressed in cancer pathways and show complex and varied mechanisms involved in CRPC, making drug targeting challenging.

In the human PCa tumour dataset, *BCL-2* is up-regulated and is known to block chemotherapy induced apoptosis [53]. TGF-β signalling may also be responsible for CRPC in the human tumour samples. TGF-β signalling is a pathway that has dual roles, tumour-suppressor and tumour-promoter in cancer depending on the cellular context and clinical stage of the disease [54]. In advanced PCa, TGF-β signalling promotes tumour progression, angiogenesis, invasiveness and epithelial-mesenchyme transition (EMT) [55]. AR and TGF-β signalling mediate EMT and the crosstalk between these two signalling pathways determines apoptotic effects in PCa [56–58].

The MAPK and PI3K-Akt pathways are highly enriched with differentially expressed genes in the cell line and the human tumour datasets. The MAPK and PI3K-Akt pathways are one of the most deregulated growth factor receptor signalling pathways in cancers [59]. These two pathways have been implicated in hormonal treatment resistance both in breast [60, 61] and prostate [27, 62] cancers and are significantly enriched pathways in our datasets. The crosstalk between growth factor receptor pathways and ER signalling that activates downstream PI3K-Akt and in turn affects proliferation and survival exists in breast cancer [63]. Likewise, in PCa, ERα drives proliferation via MAPK and PI3K signalling in PTEN-null tumours [64]. As for AR, there is evidence of crosstalk with the PI3K-Akt pathway in both androgen dependent and androgen independent PCa cells [65, 66]. In addition, the signalling pathway through ERK1/2- and MEK, which is significantly reduced in our cell line data and human clinical data, has been shown to regulate PGR function [67]. Besides having nuclear receptor signalling roles, PGR is also able to indirectly activate key signalling pathways, especially, MAPK and PI3K-Akt [68, 69]. These findings further strengthen the expression balance of SHR, which ultimately can synergistically enhance other proliferative/survival pathways in CRPC.

## Conclusions

Mechanisms driving CRPC are highly complex and diverse, and there is evidence for genetic differences across different populations [70] and numerous oncogenes and tumour suppressors are active in CRPC bypassing AR signalling. The nuclear receptor family, in particular, the steroid hormone receptor subfamily genes such as ER, PGR and GR may be compensating for the lack of AR activity to promote cell proliferation in CRPC. These genes may therefore represent potential drug targets in CRPC [71]. Our study also indicates that altered signalling driving cell proliferation and resistance to apoptosis is found in the LNCaP-AI cells, highlighting in particular a role for MAPK and PI3K signalling in driving proliferation, and the known links between PGR and MAPK signalling further reinforce this interpretation. Thus, our model system shows a complex, multi-layered response to prolonged androgen deprivation, and, consistent with data from human tumours, suggests that the combination of reprogrammed gene regulation compensating for the loss of AR activity and altered proliferative signalling drives the androgen resistant phenotype in this model.

## Availability of supporting data

Supporting data for this paper are available in the supplementary data files referenced throughout the text.

## Ethics approval

This study involved cell-based experiments and previously published publicly available data. Ethics approval was not required.

## Additional file

Additional file 1: **Table S1. ** Top 15 pathways for the human differentially expressed genes. **Table S2.** Top 15 pathways for the cell line deferentially expressed genes. **Figure S1.** Differencially expressed cell line genes overlaid on KEGG hsa04010 MAPK signaling pathway. **Figure S2.** Differentially expressed cell line genes overlaid on KEGG hsa04151 PI3K-Akt signaling pathway-Homo sapiens (human). **Figure S3.** Differentially expressed human tumour genes overlaid on KEGG hsa04010 MAPK signaling pathway. **Figure S4.** Differentially expressed human tumour genes overlaid on KEGG hsa04151 PI3K-Akt signaling pathway-Homo sapiens (human). (PDF 460 kb)

## Abbreviations

ADT: androgen deprivation therapy
Akt: protein kinase B
AR: androgen receptor
cDNA: complementary DNA
CRPC: castrate resistant prostate cancer
CS-FBS: charcoal-stripped foetal bovine serum
DBD: DNA-binding domain
DHT: dihydrotestosterone
EMT: epithelial-mesenchyme transition
ER: oestrogen receptor
GR: glucocorticoid receptor
LBD: ligand- binding domain
LHRH: luteinizing hormone-releasing hormone
MAPK: mitogen-activated protein kinase
NR: nuclear receptor
PCa: prostate cancer
PET: polyethylene terephthalate
PGR: progesterone receptor
PI3K: phosphoinositide 3-kinase
PPI: protein-protein interaction
PSA: prostate specific antigen
RNA seq: RNA sequencing
SHR: steroid hormone receptor
