# Supplementary material for: Network analysis of an in vitro model of androgen-resistance in prostate cancer
Source: BMC Cancer. 2015 Nov 10;15:883. doi: 10.1186/s12885-015-1884-7 (PMC4640359; doi:10.1186/s12885-015-1884-7)
Supplement: Additional file 1: — Table S1. Top 15 pathways for the human differentially expressed genes. Table S2. Top 15 pathways for the cell line deferentially expressed genes. Figure S1. Differencially expressed cell line genes overlaid on KEGG hsa04010 MAPK signaling pathway. Figure S2. Differentially expressed cell line genes overlaid on KEGG hsa04151 PI3K-Akt signaling pathway-Homo sapiens (human). Figure S3. Differentially expressed human tumour genes overlaid on KEGG hsa04010 MAPK signaling pathway. Figure S4. Differentially expressed human tumour genes overlaid on KEGG hsa04151 PI3K-Akt signaling pathway-Homo sapiens (human). (PDF 460 kb) [file 12885_2015_1884_MOESM1_ESM.pdf]

Top 15 Pathways Human (No of genes mapped out of 3959)
hsa01100 Metabolic pathways - Homo sapiens (human) (399)
hsa05200 Pathways in cancer - Homo sapiens (human) (119)
hsa04151 PI3K-Akt signaling pathway - Homo sapiens (human) (92)
hsa04141 Protein processing in endoplasmic reticulum - Homo sapiens (human) (73)
hsa05205 Proteoglycans in cancer - Homo sapiens (human) (67)
hsa04014 Ras signaling pathway - Homo sapiens (human) (67)
hsa04510 Focal adhesion - Homo sapiens (human) (65)
hsa04144 Endocytosis - Homo sapiens (human) (64)
hsa04015 Rap1 signaling pathway - Homo sapiens (human) (62)
hsa05166 HTLV-I infection - Homo sapiens (human) (62)
hsa04024 cAMP signaling pathway - Homo sapiens (human) (61)
hsa04810 Regulation of actin cytoskeleton - Homo sapiens (human) (59)
hsa04080 Neuroactive ligand-receptor interaction - Homo sapiens (human) (59)
hsa04010 MAPK signaling pathway - Homo sapiens (human) (58)
hsa04020 Calcium signaling pathway - Homo sapiens (human) (55)

Table 1 - Top 15 Pathways for the Human differentially expressed genes

Top 15 Pathways Cell Line (No of genes mapped out of 2611)
hsa01100 Metabolic pathways - Homo sapiens (human) (196)
hsa05200 Pathways in cancer - Homo sapiens (human) (88)
hsa03010 Ribosome - Homo sapiens (human) (77)
hsa04151 PI3K-Akt signaling pathway - Homo sapiens (human) (65)
hsa05016 Huntington's disease - Homo sapiens (human) (56)
hsa05166 HTLV-I infection - Homo sapiens (human) (49)
hsa04810 Regulation of actin cytoskeleton - Homo sapiens (human) (48)
hsa04510 Focal adhesion - Homo sapiens (human) (47)
hsa04014 Ras signaling pathway - Homo sapiens (human) (46)
hsa05010 Alzheimer's disease - Homo sapiens (human) (45)
hsa04015 Rap1 signaling pathway - Homo sapiens (human) (44)
hsa04932 Non-alcoholic fatty liver disease (NAFLD) - Homo sapiens (human) (42)
hsa04010 MAPK signaling pathway - Homo sapiens (human) (42)
hsa00190 Oxidative phosphorylation - Homo sapiens (human) (40)
hsa05012 Parkinson's disease - Homo sapiens (human) (40)

Table 2 - Top 15 Pathways for the Cell line differentially expressed genes

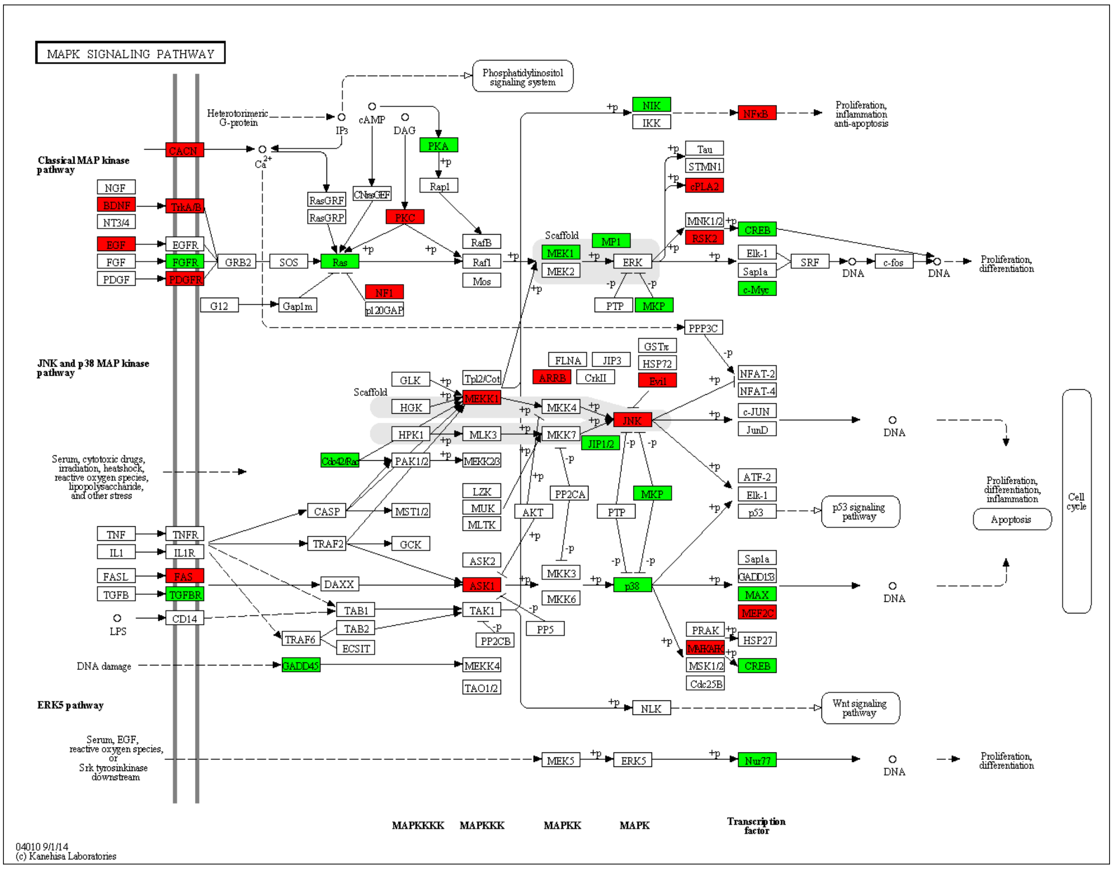

Figure 1 – Differentially expressed cell line genes overlaid on KEGG hsa04010 MAPK signaling pathway

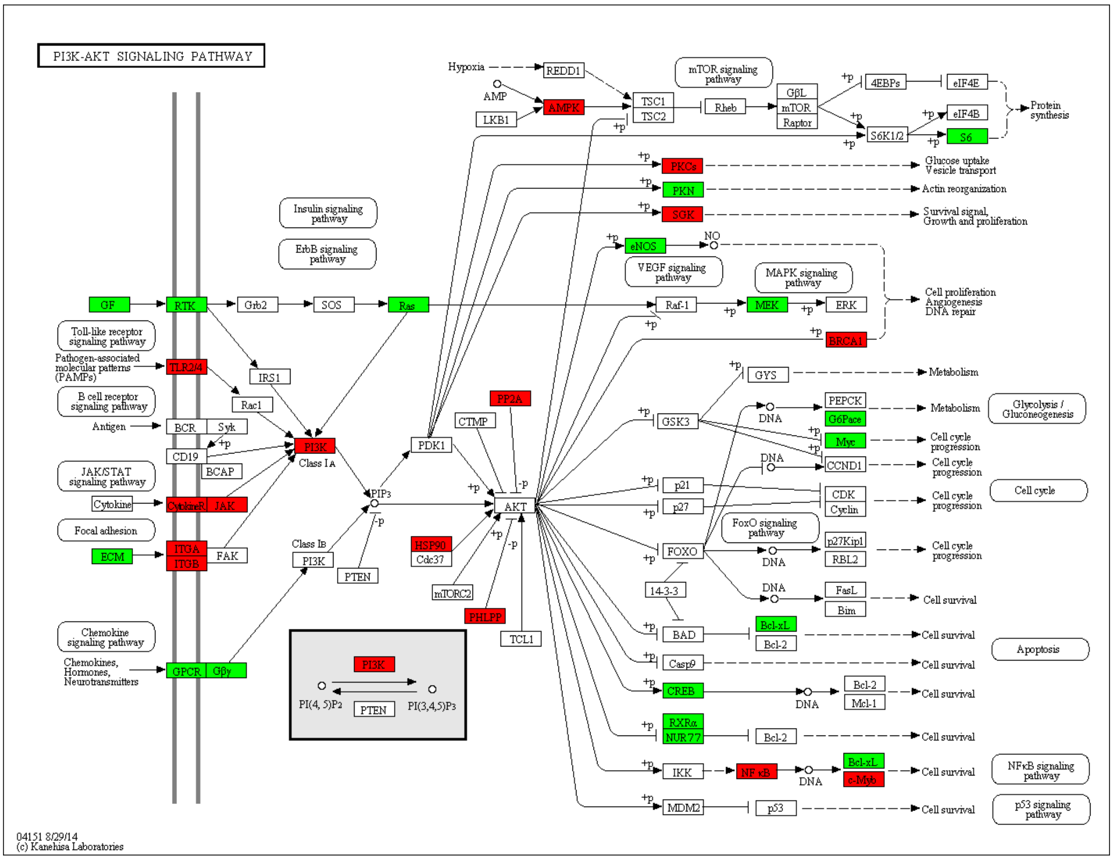

Figure 2 - Differentially expressed cell line genes overlaid on KEGG hsa04151 PI3K-Akt signaling pathway - Homo sapiens (human)

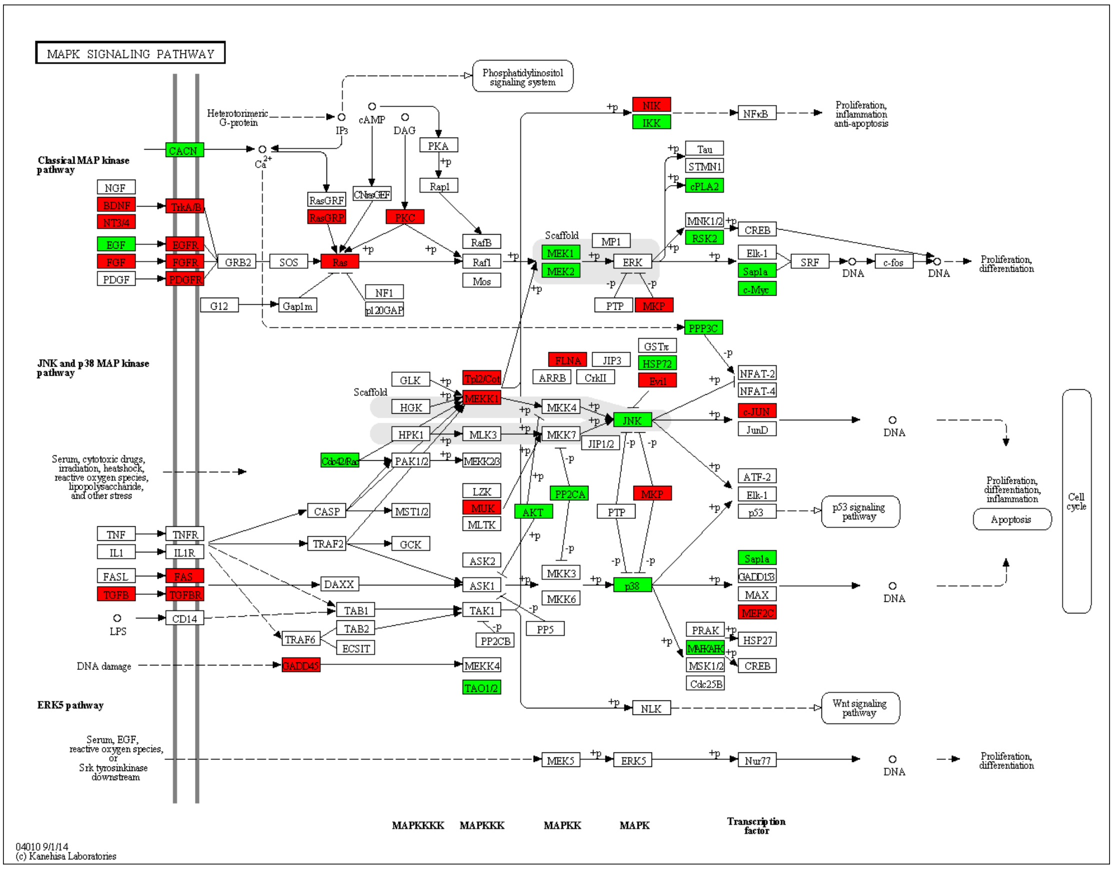

Figure 3 – Differentially expressed human tumour genes overlaid on KEGG hsa04010 MAPK signaling pathway

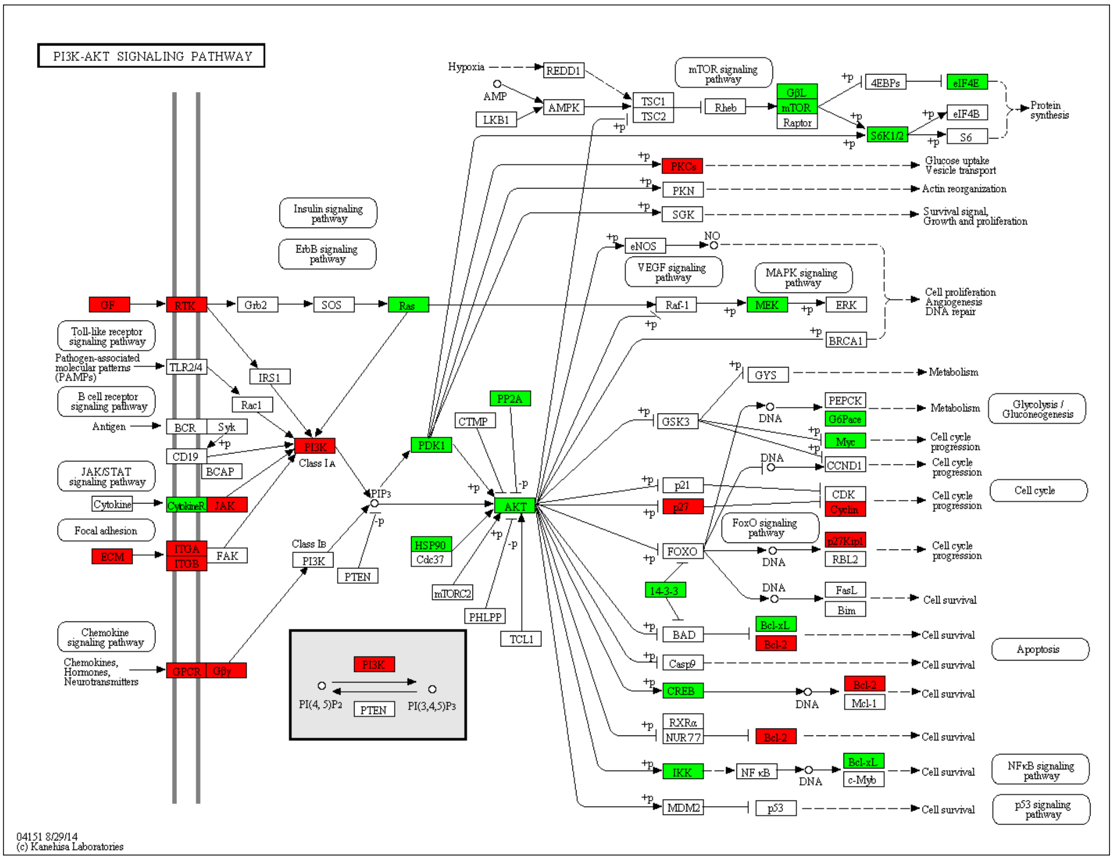

Figure 4 - Differentially expressed human tumour genes overlaid on KEGG hsa04151 PI3K-Akt signaling pathway - Homo sapiens (human)
